# Dance for Chronic Pain Conditions: A Systematic Review

**DOI:** 10.1093/pm/pnac092

**Published:** 2022-06-23

**Authors:** Benjamin Hickman, Fereshteh Pourkazemi, Roxanna N Pebdani, Claire E Hiller, Alycia Fong Yan

**Affiliations:** Faculty of Medicine and Health, The University of Sydney, Sydney, NSW, Australia; Faculty of Medicine and Health, The University of Sydney, Sydney, NSW, Australia; Faculty of Medicine and Health, The University of Sydney, Sydney, NSW, Australia; Faculty of Medicine and Health, The University of Sydney, Sydney, NSW, Australia; Faculty of Medicine and Health, The University of Sydney, Sydney, NSW, Australia

**Keywords:** Dance for Health, Fibromyalgia, Pain Management, Pain Experience, Dance Therapy

## Abstract

**Objectives:**

Globally, 20–25% of people will experience chronic pain in their lifetimes. Dance is a physical activity with psychosocial benefits that might positively impact pain. This review aimed to investigate the effect of dance interventions on the experience of pain by quantitative measures and qualitative themes.

**Methods:**

Seven major databases were searched from inception to January 2021. Two independent reviewers screened articles at each stage. Qualitative and quantitative studies were included if the dance interventions lasted more than 6 weeks, participants reported pain of duration longer than 3 months, and pain was an outcome of the study. All articles were critically appraised with appropriate Joanna Briggs Institute tools, and data were collated through the use of results-based convergent synthesis.

**Results:**

From 23,628 articles, 34 full papers were included, with a total of 1,254 participants (75.2% female). Studies predominantly investigated individuals with fibromyalgia (26%) and generalized chronic pain (14%), with aerobic dance (20.7%) and Biodanza (20.7%) being the most common dance genres investigated. Overall, 74% of studies noted either reduced pain through quantitative pain measures or qualitative themes of improved pain experience (88% for chronic primary pain and 80% for chronic secondary musculoskeletal pain).

**Discussion:**

There were positive effects of dance on chronic primary and secondary musculoskeletal pain across diverse populations. A variety of study designs and interventions noted improved pain measures and themes around pain coping and acceptance, with all dance therapies showing improvements, particularly when performed for 60–150 minutes’ duration weekly. Dance should be considered as an effective adjunct in the management of chronic pain.

## Introduction

Pain that persists into chronicity is a common and challenging phenomenon, as it is multifaceted and could require several treatment modalities to be managed effectively [1]. Pain is defined as “an unpleasant sensory and emotional experience associated with, or resembling that associated with, actual or potential tissue damage” [2], with chronic pain defined as persistent or recurrent pain lasting for longer than 3 months [3]. Chronic pain is now considered a clinical disease in itself and involves a complex interplay among biological, psychological, and social factors [4]. Recent classifications of pain have differentiated types of chronic pain, notably chronic primary and chronic secondary pain syndromes, and have noted differences in diagnosis and characteristics [5]. One common presentation, chronic primary pain, may be defined as a pain syndrome of longer than 3 months’ duration that cannot be accounted for by another pain condition, and secondary musculoskeletal pain may be defined as pain that arises from a disease process that affects the musculoskeletal system [5]. Chronic pain is associated with heightened stress responses [6], deconditioning [7], fear and catastrophization [8], and feelings of separation and isolation [9]. Additionally, over time, there is the potential for increased sensitivity of the nervous system through pathophysiological changes [10]. Therefore, a biopsychosocial approach to chronic pain management, which considers the multifactorial nature and dynamic interaction of human functioning and the unique pain experience of each individual, has been widely adopted [4, 11].

Evidence on multidisciplinary pain management approaches has demonstrated improvements in quality of life in those with fibromyalgia [12], improved pain levels in complex chronic pain conditions [13], and greater efficacy than isolated physical therapy treatments alone [14]. Coordinating multimodal treatment is necessary for the best outcome for those experiencing chronic pain. Therefore, interventions that promote physical activity, self-efficacy [15, 16], and social connection [17] are important in the management of chronic pain conditions [18, 19].

Current pain management practices emphasize the need for physical activity that reintroduces activities needed for daily living [20], addressing maladaptive beliefs [21], and using social connection as a means of reducing symptoms and improving quality of life [22]. Frequent activity for people experiencing chronic pain could aid in reducing pain and related symptoms [23]. Additionally, therapies with high adherence and progressive exposure to activity that assists in finding active coping strategies are beneficial [24]. The use of graded activity or graded exposure could be helpful in reducing pain intensity [25], improving quality of life and reducing disability in the long term [24], and catastrophization in the short term [26]. Other strategies of activity pacing could assist in pain coping with an emphasis on meaningful activities [27]. Therefore, the use of dance could fit into the biopsychosocial model of health care.

Dance is defined as “a series of steps and movements that match the speed and rhythm of a piece of music” [28]. Dance can be described in many ways, most commonly by genre (for example, ballet, ballroom, hip-hop), and categorized into the contexts of performance, competition, social dancing, or dance therapy. These contexts differ by the motivation and goals of dancing, of which performance and competitive dancing requires more hours of training, psychosocial stress [29], and attention to technique. In contrast, dance in a social setting is structured around a particular genre and performed for recreational purposes with a partner or in a group setting. These genres of dancing are considered as “structured dances” in the context of the present article. In comparison, dance can also be used a form of therapy, in which there is no structure per se in how it may be performed but rather an emphasis on creation and exploration of movement and music.

Unstructured dance can be used for the purpose of addressing an identified issue, which may be termed “dance therapy,” or it can have the aim of creating and improvising a dance to a given piece of music, generally defined as a “creative dance.” Two popular genres of dance therapy are Biodanza and Dance Movement Therapy (DMT). Biodanza is defined as “an intervention intended to promote health by encouraging self-expression and autoregulation through music, dance and interaction” [30]. DMT is defined as “the use of creative movement and dance in a therapeutic relationship” [31]. Both creative dances and dance therapies are grounded in exploration of movement and music and an interoception of the body.

Dance has a range of benefits that address the physical and biological issues associated with a number of health conditions. Research investigating dance and health found that a variety of dance genres showed improved body composition, blood biomarkers, and musculoskeletal function [32]. Dance has also been shown to improve pain, quality of life, impact of disease, and function in those with fibromyalgia [33]. Other physiological benefits of dance include improvements in cardiovascular parameters, balance, and stride velocity [34]. When dance interventions are compared with other forms of exercise, it appears dance has an equal, and at times superior, effect on physical health benefits [32].

Although dance has many physical benefits, these do not occur without the presence of psychosocial benefits. Dance promotes psychosocial benefits such as socialization [35], in-group bonding, [36] eye contact [37], and touch [38], which in turn appear to improve mood and self-confidence [35] and pain thresholds [36]. Dance has significantly reduced the effects of depression and anxiety, and it improves confidence in ability to cope with serious mental illness [39]. Additionally, improvements in health-related quality of life, mental representations linked to body image, and consciousness have also been noted in obese individuals participating in dance therapy [40]. When compared with exercise alone, dance might have the most significant effects on depression in those with psychiatric disorders [41]. Therefore, dance has the potential to address the wider psychosocial issues that people may be experiencing.

Given the numerous benefits of dance from a biopsychosocial context, the use of dance could address the multifactorial nature of chronic pain conditions, with the potential to have increased adherence [42] compared with conventional guided exercises [32]. This could be due to the reported experiences of joy, satisfaction, and increased motivation [43] associated with dance. Additionally, the use of music could be an important aspect of adherence to and enjoyment of a dance intervention. A 2006 Cochrane Review [44] on music for pain relief found small positive effects on pain reduction and reduced requirements for analgesics. Music could also be a beneficial adjunct in chronic pain management when self-selected [45], with increased pain thresholds when there is an active component such as dancing [46] that is more potent than just exercise alone [47]. As dance is a highly adaptable form of activity that can be modified to different physical and cognitive loads, it offers an enjoyable form of graded activity and pacing strategy [48].

It is evident that dance can have a broad range of benefits that address the physical, cognitive [49], and psychological issues [50] associated with a number of clinical conditions. However, a wider consensus on dance for chronic pain management, via a mixed-methods synthesis, and recommendations for dance interventions are lacking and constitute a gap in the current literature. Investigating the consensus of quantitative and qualitative literature will aid in gaining a wider view of the multifactorial nature of pain. In addition, there is typically a mismatch within patient-directed care, wherein patients prioritize pain reduction and management and clinicians prioritize improving function [51]. Therefore, the aim of the present systematic review was to investigate the effect of dance interventions on pain perception through quantitative pain outcome measures and through psychosocial benefits identified by qualitative themes related to pain experience. We also endeavored to provide practical recommendations for dance interventions. We hypothesized that dance would have positive effects in reducing the perception of pain and have indirect psychosocial benefits in populations experiencing chronic pain.

## Methods

This systematic review was registered in the International Prospective Register of Systematic Reviews (CRD42020165557) [52] and adhered to the Preferred Reporting Items for Systematic Reviews and Meta-Analysis (PRISMA) guidelines [53].

Initial preliminary database searches were performed via Medline and Embase to determine the most suitable key terms required to address the research questions. The final search was performed via seven electronic databases, including Medline, Embase, Web of Science, Scopus, CINAHL, SportsDiscus, and AMED from earliest records until February 1, 2021. The search strategy included two domains, one involving general dance key terms and dance genres and the second involving general key terms around pain, treatment, and therapy. Search terms from the dance domain and pain domain were combined with Boolean operator “OR,” and the two domains were combined with “AND” (Table 1). This strategy was designed in conjunction with a specialized health sciences librarian.

**Table 1.** Database search strategy used

1. Dance			2. Pain
Dancing/ Danc*.mp Couple danc*.mp Social danc*.mp Partner* danc*.mp Group danc*.mp Dance sport*.mp Ballroom danc*.mp Latin danc*.mp Biodanza*.mp Dance therap*.mp Resseguier*.mp Free dance movement*.mp Exercise Movement Technique*.mp Creative danc*.mp Rueda*.mp Contemporary danc*.mp Rumba*.mp	Tango*.mp Semba*.mp Samba*.mp Bellydance*.mp Cha Cha*.mp Waltz*.mp Irish danc*.mp Cultural danc*.mp Africa* danc*.mp Disco danc*.mp Electronic danc*.mp Rhythm danc*.mp Street danc*.mp Swing danc*.mp Hip hop danc*.mp Bachata*.mp Forro*.mp Salsa*.mp Mambo*.mp Improvisation*.mp Modern danc*.mp	Tap danc*.mp Jazz danc*.mp Free danc*.mp Dance improvisation.mp Interpretive danc*.mp Ballet.mp Medieval danc*.mp Circle danc*.mp Line danc*.mp Round danc*.mp Square danc*.mp Calypso*.mp Flamenco*.mp Zumba*.mp Argentine tango*.mp Danza*.mp Jive*.mp Merengue*.mp Dance movement therap*.mp	Pain/ Pain*.mp Pain management/ Pain relief*.mp Pain control*.mp Treat*.mp Therapeutics/ Therap*.mp

Search strategy included one term from the Dance column and one from the Pain column.

Articles were imported into EndNote (Clarivate Anytics, Philadelphia, PA, Version X9) [54] for duplicate removal, after which they were imported into the data management software Covidence (Covidence systematic review software, Veritas Health Innovation, Melbourne, Australia. Available at www.covidence.org) [55]. Articles were independently screened against the eligibility criteria by two independent reviewers (BH with AFY, FP, RP, or CH) via title and abstract. Eligibility criteria were as follows: human population, pain as an outcome measure, chronic pain of at least 3 months’ duration, dance intervention of 6 weeks or longer, and dance that included music and movement (Table 2). Studies were excluded if they studied acute pain (defined as less than 3 months’ duration), were reviews or non-experimental evidence, or if they studied music therapy, art therapy, Pilates, or yoga.

**Table 2.** Eligibility criteria

Inclusion Criteria	Exclusion Criteria	
Human population Pain as outcome measure Chronic pain (≥3 months) Dance intervention of ≥6 weeks Dance must include music and movement	Animal population Acute pain (<3 months) Books Excepts Opinion Abstracts Systematic reviews	Music therapy Art therapy Pilates Yoga

Used during the screening process of abstracts and full articles.

Data were extracted from full texts by two independent reviewers (BH with AFY, FP, RP, or CH) using a pre-piloted extraction form. Any conflicts were resolved via group discussion. Data extracted included study years, populations, age range, location, dance intervention details, compliance, dropouts, post-intervention follow-up length, pain outcome measures such as subjective scales, pain-related outcome measures within questionnaires and questionnaire total score, and major qualitative themes relating to pain experience. Compliance was defined as the number of sessions attended by the participants and dropouts as the number of participants who did not finish the intervention. For studies that had a progressive increase in duration of dance sessions, the duration was determined by the duration of dance performed by the end of the study.

Included articles were assessed for risk of bias at the study level through the use of the Joanna Briggs Institute (JBI) critical appraisal tools for Randomized Controlled Trials [56], Quasi-Experimental Studies [56], Qualitative Studies [57], and Case Series [58] (Figures 2A–D). Mixed-methods studies were assessed with both the respective quantitative and qualitative JBI checklists. Two reviewers (BH with AFY, FP, RP, or CH) assessed each article and resolved conflicts within each of the checklists, with disputes being resolved via group discussion among the five reviewers involved.

Studies were categorized into chronic pain categories via the *International Classification of Diseases,11^th^ Revision* [3]. Studies were also categorized as either structured dance or dance therapy. Quantitative study data were planned to be synthesized via a random-effects meta-analysis with pain as the outcome measure if appropriate [59]. Qualitative study data have been presented as a results-based convergent synthesis [60] reporting on the main themes of pain and the participant’s changed relationship to pain.

## Results

**Figure 1.**
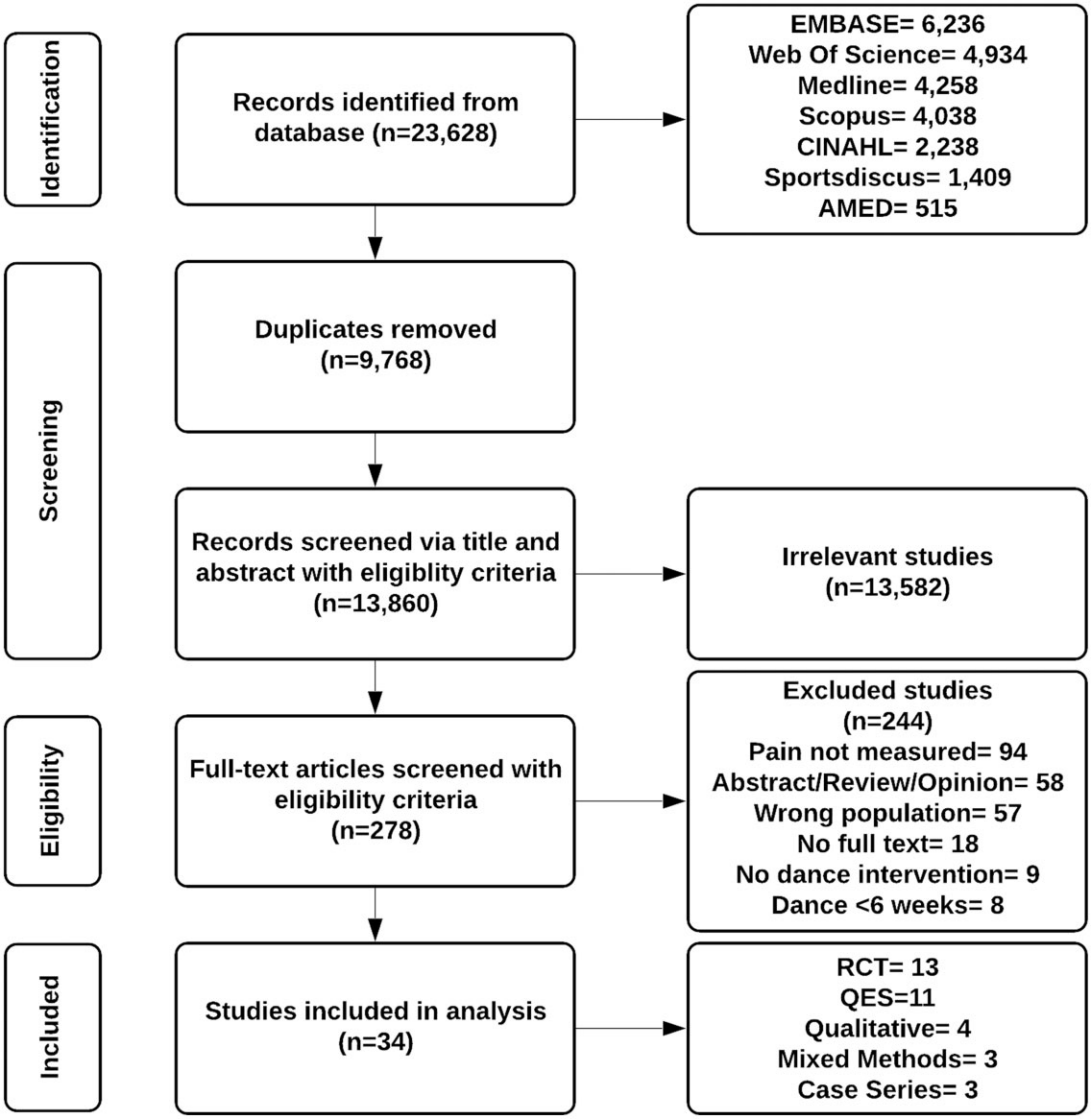
PRISMA flow chart.

The initial search yielded 23,628 articles. After screening, 34 articles were included for review (Figure 1). Meta-analysis was considered inappropriate because of the heterogeneity of the data, and as such, a narrative synthesis of both the quantitative and qualitative data has been reported. This review identified 27 quantitative studies, four qualitative studies [61–64], and three mixed-methods studies [65–67]. The quantitative studies consisted of 13 randomized controlled trials [68–80], 11 quasi-experimental studies [81–91], and three case series [92–94]. One randomized controlled trial produced two articles [70, 71], and one quasi-experimental study produced two articles [82, 90] with different measures reported. For qualitative studies, the theoretical framework was not specified, and data were collected via interviews [61, 63, 64] or focus groups [62].

Most participants in the studies were women (n = 943 women; 75.2% of the included population) with an age range of 10–99 years, with 76.5% of participants 45–70 years of age. Of the studies in this review, only four noted that the participants had previous dance experience, but they did not elaborate on the participants’ ability or the extent of their experience. The most studied populations included those with fibromyalgia [68, 70, 71, 75–77, 81–83, 85, 90] and those with nonspecific diagnoses of chronic or persistent pain [63, 64, 67, 94] or medically undiagnosed symptoms [63, 64, 66, 67, 94]. On the basis of classifications of pain according to the *International Classification of Diseases,11^th^ Revision* [5], pain conditions in 15 studies were classified as chronic primary pain [63, 64, 66–68, 70, 71, 75–77, 81–83, 85, 90, 94], with 10 of those classified as chronic secondary musculoskeletal pain [61, 69, 72–74, 78, 79, 87–89], three as chronic neuropathic pain [84, 91, 93], two as chronic cancer treatment pain [62, 86], and three as chronic primary pain [65, 80, 92]. Further details on participant demographics and pain classifications can be found in Table 3.

**Table 3.** Demographic summary of participants and classifications of their diagnosed conditions

Study	Population (Total Number)	ICD-11 Classification	Age, years (Mean ± Standard Deviation or Range)	Gender (F:M)
Baptista et al., 2012	Fibromyalgia (80)	Chronic primary	49.3	80F
Barene et al., 2014	Hospital employees (107)	Chronic secondary MSK	45.8 ± 9.3	107F
Bojner Horwitz et al., 2006	Fibromyalgia (36)	Chronic primary	57 ± 7.2	36F
Bojner Horwitz et al., 2003	Fibromyalgia (36)	Chronic primary	57 ± 7.2	36F
Broscheid et al., 2020	Lower back pain with spinal stenosis and neurogenic claudication (32)	Chronic primary MSK	70 ± 10.6	24F, 8M
Casilda-Lopez et al., 2017	Postmenopausal women with knee OA (34)	Chronic secondary MSK	65.6 ± 7.2	34F
Kaholokula et al., 2017	Hypertensive Pacific Islanders (53)	Chronic secondary MSK	55 ± 10	47F, 6M
Krampe et al., 2014	Older adults with leg pain (37)	Chronic secondary MSK	80 ± 8.9	31F, 6M
Lopez-Rodriguez et al., 2012	Fibromyalgia (70)	Chronic primary	55.4 ± 7.5	70F
Lopez-Rodriguez et al., 2013	Fibromyalgia (76)	Chronic primary	54.8 ± 7.8	76F
Norregaard et al., 1997	Fibromyalgia (38)	Chronic primary	50	UR
Qin et al., 2018	Postmenopausal women with osteoporosis (50)	Chronic secondary MSK	45–60	50F
Tharani et al., 2018	Primary dysmenorrhea (30)	Chronic secondary MSK	17–23	30F
Assunçao Júnior et al., 2017	Fibromyalgia (25)	Chronic primary	52.6	25F
Carbonell Baeza et al., 2010	Fibromyalgia (71)	Chronic primary	54 ± 6.2	71F
Carbonell Baeza et al., 2012	Fibromyalgia (38)	Chronic primary	MD 50.9 ± 7.7 Biodanza 54.5 ± 7.5	38F
Cherriere et al., 2020	Charcot-Marie-Tooth Disease hereditary peripheral neuropathy type (9)	Chronic neuropathic	10.2 ± 1.5	7F, 2M
De Carvalho et al., 2012	Hemiparetic stroke (8)	Chronic neuropathic	Female (58.2 ± 3.8) Male (64.3 ± 5.9)	5F, 3M
Maddali Bongi et al., 2012	Fibromyalgia (38)	Chronic primary	57.3 ± 11.5	UR
Mirandola et al., 2015	Breast cancer survivors (18)	Chronic cancer treatment	53 ± 7.7	18F
Moffet et al., 2000	Rheumatoid arthritis (10)	Chronic secondary MSK	54 ± 10	10F
Noreau et al., 1995	Rheumatoid arthritis I/II (29)	Chronic secondary MSK	49.3 ± 13	20F, 9M
Perlman et al., 1990	Rheumatoid arthritis (43)	Chronic secondary MSK	40–60: 51% >60: 33%	41F, 2M
Segura-Jimenez et al., 2017	Fibromyalgia (27)	Chronic primary	54.2 ± 6.2	27F
Beerenbrock et al., 2019	Parkinson’s disease (12)	Chronic secondary MSK	67.1	10F, 11M
Crane- Okada et al., 2012	Breast cancer survivors (49)	Chronic cancer treatment	66.3	49F
Flanagan, 2004	Chronic pain (153)	Chronic primary	45	UR
Nordstrom et al., 2018	Persistent pain (20)	Chronic primary	UR	19F, 1M
Okafor et al., 2012	Nonspecific low back pain (30)	Chronic primary MSK	55.2	20F, 10M
Payne, 2009	Chronically medically undiagnosed symptoms (24)	Chronic primary	UR	UR
Shim et al., 2017	Chronic pain (22)	Chronic primary	51.9 ± 8.8	16F, 6M
Castrillon et al., 2017	Chronic lower back pain (2)	Chronic primary MSK	22.5	2F
Ribeiro et al., 2011	Multiple sclerosis (3)	Chronic neuropathic	45	2F, 1M
Simoes et al., 2020	Institutionalized older adults (7)	Chronic primary	86 (mean) 68–99	5F, 2M
Total studies: 34 articles 32 studies	Total participants = 1,254 Fibromyalgia = 9 (446) Chronic/persistent pain = 5 (226) Rheumatoid arthritis = 3 (82) Breast cancer patients = 2 (67) Lower back pain = 3 (64)	Chronic primary = 16 Chronic secondary MSK = 10 Chronic neuropathic = 3 Chronic cancer treatment = 2 Chronic primary MSK = 3	45–70 = 26 <45 = 3 >71 = 1 Unclassifiable = 4	F = 943 (75.2%) M = 67

ICD-11 = *International Classification of Diseases, 11^th^ Revision*; F= female; M= male; MSK = musculoskeletal; MD = multidisciplinary; OA = osteoarthritis; UR = unreported.

The most common dance interventions were aerobic dance (17.6%), Biodanza (17.6%), DMT (11.8%), and choreographed dances (11.8%), about which varied details were given about the structure across the interventions (Table 4). Aerobic dance [65, 77, 79, 87–89] generally involved various types of movement coordinated with higher-tempo music, but details about the intervention were poorly described and had missing or questionable data for analysis [65, 77, 79]. Types of dance were classified into 20 structured dance interventions [61, 65, 68, 69, 73, 74, 77–80, 84–89, 91–94] and 14 dance therapies [62–64, 66, 67, 70–72, 75, 76, 81–83, 90]. The dance therapies were all exploratory in nature and involved creative components that allowed self-expression and improvisation. All dance interventions were performed in a group setting but done individually, with the exception of one study that utilized a partnered tango dance [61]. All dances were also led by a dance instructor who facilitated each session, with no studies detailing the use of mirrors in class.

**Table 4:** Summary of interventions and outcomes for included articles

Study	Study Design	Dance Intervention	Comparator	Pain Measure (Statistical Significance)	Follow-Up (SS)	Compliance, %	Dropouts, %
Baptista et al., 2012*	RCT	**Belly dance** 60 minutes ×2/week for 16 weeks	Waitlist (40) Dance (40)	VAS SF36 (SS)	16 weeks VAS (SS)	83	5
Barene et al., 2014*	RCT (Cluster)	**Zumba** 60 minutes ×2–3/week for 40 weeks	Soccer (37) Zumba (35) Control (34)	Nordic MSK Questionnaire (SS)	None	84	14.3
Bojner Horwitz et al., 2003*	RCT	**DMT** 60 minutes ×1/week for 24 weeks	Control (16) Dance (20)	VAS	None	UR	UR
Bojner Horwitz et al., 2006*	RCT	**DMT** 60 minutes ×1/week for 24 weeks	Control (16) Dance (20)	VAS GAWP (SS)	32 weeks GAWP (SS)	UR	UR
Broscheid et al., 2020	RCT	**Choreographed dance** 60 minutes ×2/week for 6 weeks	Multimodal intervention (14) Physio control (10)	Brief Pain Inventory Oswestry Low Back Pain Index	None	>80	UR
Casilda-Lopez et al., 2017	RCT	**Biodanza** 45 minutes ×3/week for 8 weeks	Control (17) Biodanza (17)	WOMAC (SS)	12 weeks	UR	0
Kaholokula et al., 2017	RCT	**Hula dance** 60 minutes ×2/week for 12 weeks	Waitlist (28) Dance (27)	SF12	12 weeks	87	7.4
Krampe et al., 2014	RCT	**Dance** 45 minutes ×2/week for 12 weeks	Waitlist (15) Dance (19)	Functional Pain Scale (SS)	None	88	10.5
Lopez-Rodriguez et al., 2012*	RCT	**Aquatic Biodanza** 60 minutes ×2/week for 12 weeks	Stretching (35) Dance (35)	Pressure algometry (SS) VAS (SS) SF36 FIQ (SS) MMQ (SS)	None	>58	45.7
Lopez-Rodriguez et al., 2013*	RCT	**Aquatic Biodanza** 60 minutes ×2/week for 12 weeks	Stretching (38) Dance (38)	Pressure algometry (SS) VAS (SS) SF36 FIQ (SS) MMQ (SS)	None	>60	21.1
Norregaard et al., 1997*	RCT	**Aerobic dance** 50 minutes ×3/week for 12 weeks	Control (8) Exercise (15) Dance (15)	Pressure algometry FIQ Pain Scale	None	UR	66.7
Qin et al., 2018	RCT	**Square dance** 30–60 minutes ×5/week for 24 weeks	Control (25) Dance (25)	WHO 4 Level Pain Grade (SS)	None	UR	UR
Tharani et al., 2018*	RCT	**Aerobic dance** 45 minutes ×3/week for 8 weeks	Stretch (15) Dance (15)	VAS (SS)	None	UR	UR
Assunçao Júnior et al., 2017*	QES	**Zumba** 50 minutes ×2/week for 12 weeks	–	VAS (SS) FIQ SF36	None	86	24
Carbonell Baeza et al., 2010*	QES	**Biodanza** 120 minutes ×1/week for 12 weeks	Usual care (32) Biodanza (27)	Pressure algometry (SS) FIQ (SS) SF36 (SS) VPMI (SS)	None	85.6	27.1
Carbonell Baeza et al., 2012*	QES (pre/post)	**Biodanza** 120 minutes ×1/week for 16 weeks	MDT (21) Biodanza (17)	Pressure algometry (SS) FIQ (SS) SF36 (SS) VPMI (SS)	None	85.4	23.5
Cherriere et al., 2020	QES (pre/post)	**Adapted dance** 60 minutes ×2/week for 10 weeks	Control (4) Adapted dance (5)	VAS (SS)	None	89	0
De Carvalho et al., 2012	QES (Pre-Experimental)	**Brazilian folk dance** 60 minutes ×2/week for 12 weeks	None	SF36	None	UR	UR
Maddali Bongi et al., 2012*	QES (Crossover Study)	**Resseguier** 60 minutes ×2/week (first 3 weeks) ×1/week (after 4 weeks)	RM then QG (15) QG then RM (15)	Pressure algometry RPS (SS) SF36 (SS) FIQ (SS)	12 weeks FIQ (SS)	100	21
Mirandola et al., 2015	QES (Pre-Experimental)	**Choreographed dance** 50–60 minutes ×1/week for 8 weeks	None	SF12 NRS (SS)	None	UR	0
Moffet et al., 2000	QES (Pre-Experimental)	**Aerobic dance** 60 minutes ×2/week for 8 weeks	None	RAI	None	92.5	0
Noreau et al., 1995*	QES	**Aerobic dance** 35–50 minutes ×2/week for 12 weeks	Control (10) Dance (19)	AIMS (SS) Painful joints (SS)	24 weeks	83.3	UR
Perlman et al., 1990*	QES (Pre-Experimental)	**Aerobic dance** 120 minutes ×2/week for 16 weeks	None	VAS (SS) AIMS (SS) ROM pain	None	75 (for more than 77% participants)	19
Segura-Jimenez et al., 2017*	QES	**Biodanza** 120 minutes ×1/week for 12 weeks	None	VAS (SS)	None	85.6	27
Beerenbrock et al., 2019	Qualitative	**Tango** 60 minutes ×1/week for 10 weeks	–	Interviews	None	UR	UR
Crane- Okada et al., 2012	Qualitative Study	**Mindful movement** 120 minutes ×1/week for 20 weeks	Control (19) Dance (30)	Interviews	None	52 (average)	46.7
Flanagan, 2004	Qualitative Study (Experiential)	**DMT** 120 minutes ×1/week for 9 weeks	None	Interviews	None	71.9	28.1
Nordstrom et al., 2018	Qualitative Study	**Free Dance Movement** 90 minutes for 1–6 semesters	None	Interviews	None	UR	UR
Okafor et al., 2012*	Mixed Methods (RCT + qualitative)	**Aerobic dance** 45 minutes ×3/week for 6 weeks	Physio (15) Physio + Dance (15)	VAS (SS) RMDQ Interviews	None	UR	UR
Payne, 2009*	Mixed Methods (QES Crossover + qualitative)	**BodyMind Approach** 120 minutes ×1/week for 12 weeks	None	Interviews	12 weeks	UR	25
Shim et al., 2017	Mixed Methods (QES Pre-Experimental + qualitative)	**DMT** 70 minutes ×1/week for 10 weeks	None	VAS (SS) NRS Patient journal and interviews	None	UR	13.6
Castrillon et al., 2017*	Case Series	**Belly dance** 30 minutes ×2/week for 6 weeks	None	NPRS Oswestry Low Back Pain Index	8 weeks	UR	0
Ribeiro et al., 2011	Case Series	**Choreographed dance** 90 minutes ×1/week for 36 weeks	None	SF36	None	UR	UR
Simoes et al., 2020*	Case Series	**Choreographed dance** 65 minutes ×1/week for 6 weeks	None	NRS NPQ Pain Catastrophizing Scale	None	100	0
Total Studies: 34 articles 32 studies	RCT = 13 (38.2%)QES = 11 (32.4%)Qualitative = 4 (11.8%)Mixed Methods = 3 (8.8%)Case Series = 3 (8.8%)	Aerobic Dance = 6 (17.6%)Biodanza = 6 (17.6%)Choreographed dance = 4 (11.8%)DMT = 4 (11.8%)Average duration = 69.9 minutesAverage frequency = 1.8×/weekAverage length = 13.6 weeks	No control = 13 (38.2%)	Pain-related Questionnaire = 18Short Form = 920 quantitative studies with statistically significant reduction in pain (70.4%)	Average = 17.8 weeks (9 studies)	Unreported = 15	0% = 61–25% = 1125–50% = 551–75% = 1Unreported = 11

QES= quasi-experimental study; RCT= randomized controlled trial; UR= unreported; MSK= musculoskeletal; DMT= Dance Movement Therapy; RM= Rességuier Method; QG= Qi Gong; VAS= visual analog scale; SF36= Short-Form 36; SF12= Short-Form 12; GAWP= Global Assessment of Wellbeing and Pain; FIQ= Fibromyalgia Impact Questionnaire; RA= rheumatoid arthritis; VPMI= Vanderbilt Pain Management Inventory; WOMAC= Western Ontario and McMaster Universities Osteoarthritis Index; NPRS= Numeric Pain Rating Scale; SS= statistical significance; MMQ= McGill Melzack Questionnaire; RPS= Regional Pain Scale; NRS= Numeric Rating Scale; RAI= Ritchie Articular Index; AIMS= Arthritis Impact Measurement Scale; ROM pain= articular pain on motion; RMDQ= Roland Morris Disability Questionnaire; NPQ= Neurophysiology of Pain Questionnaire.

* Studies investigating pain as a primary outcome measure.

The average intervention duration across all included studies was 69.9 minutes of dance per session, ranging from 30 to 120 minutes. Average reported frequencies of dance were 1.8 times per week, ranging from 1 to 5 times per week. Average intervention length was 13.6 weeks, ranging from 6 to 40 weeks. Structured dances tended to be shorter than 60 minutes and had a greater variety of dance genres and structures when compared with the dance therapies. The dance therapy sessions tended to be longer in duration (60–120 minutes) and largely involved participants with fibromyalgia (45.5%), along with goals of movement experimentation, play, and self-expression [63, 70, 71, 92]. Across all studies, there was large heterogeneity in comparison groups, ranging from no intervention to provision of usual care, other activities, and therapy, and there was different sequencing of interventions, with 38.2% of studies offering no control group.

Of all studies, only 58.1% investigated pain as a primary outcome measure. Quantitative pain data were measured via several tools. Twenty studies used unidimensional outcome measures, such as a visual analog scale [65, 67, 68, 70, 71, 75, 76, 79, 85, 89–91] or other numerical rating scales [67, 74, 77, 78, 81, 86, 88, 89, 92, 94] in conjunction with other pain outcome measures, with three solely using a visual analog scale [79, 90, 91]. Of the six studies using the visual analog scale, the average decrease in score was two points on a 10-point scale [68, 75, 76, 79, 85, 90]. There were 18 studies that used multidimensional measures, such as specific questionnaires that have a pain component, with the majority of these using a 36-Item Short-Form Survey (SF-36) [68, 81–85, 93] or 12-Item Short-Form Survey (SF-12)[73, 86], from which bodily pain data were extracted. One study did not report bodily pain [86] in their SF-12.

**Figure 2.**
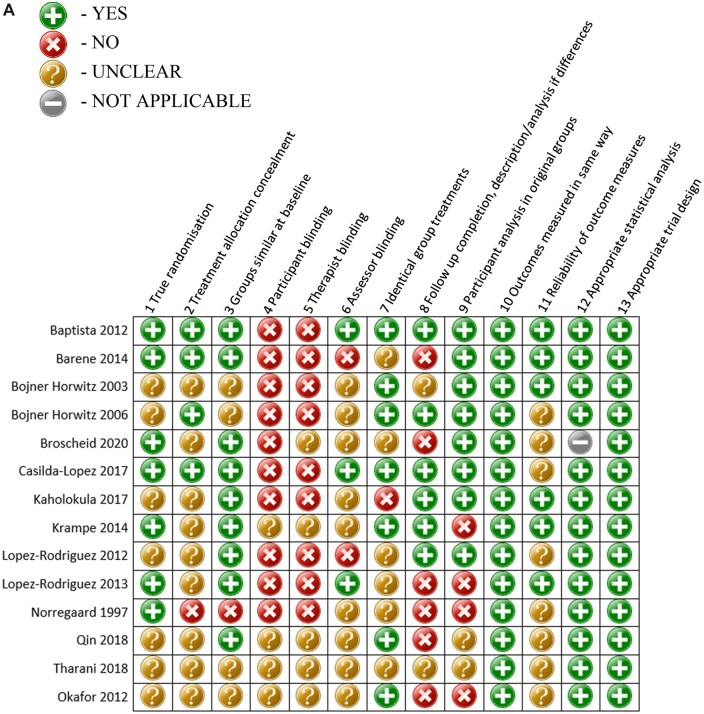

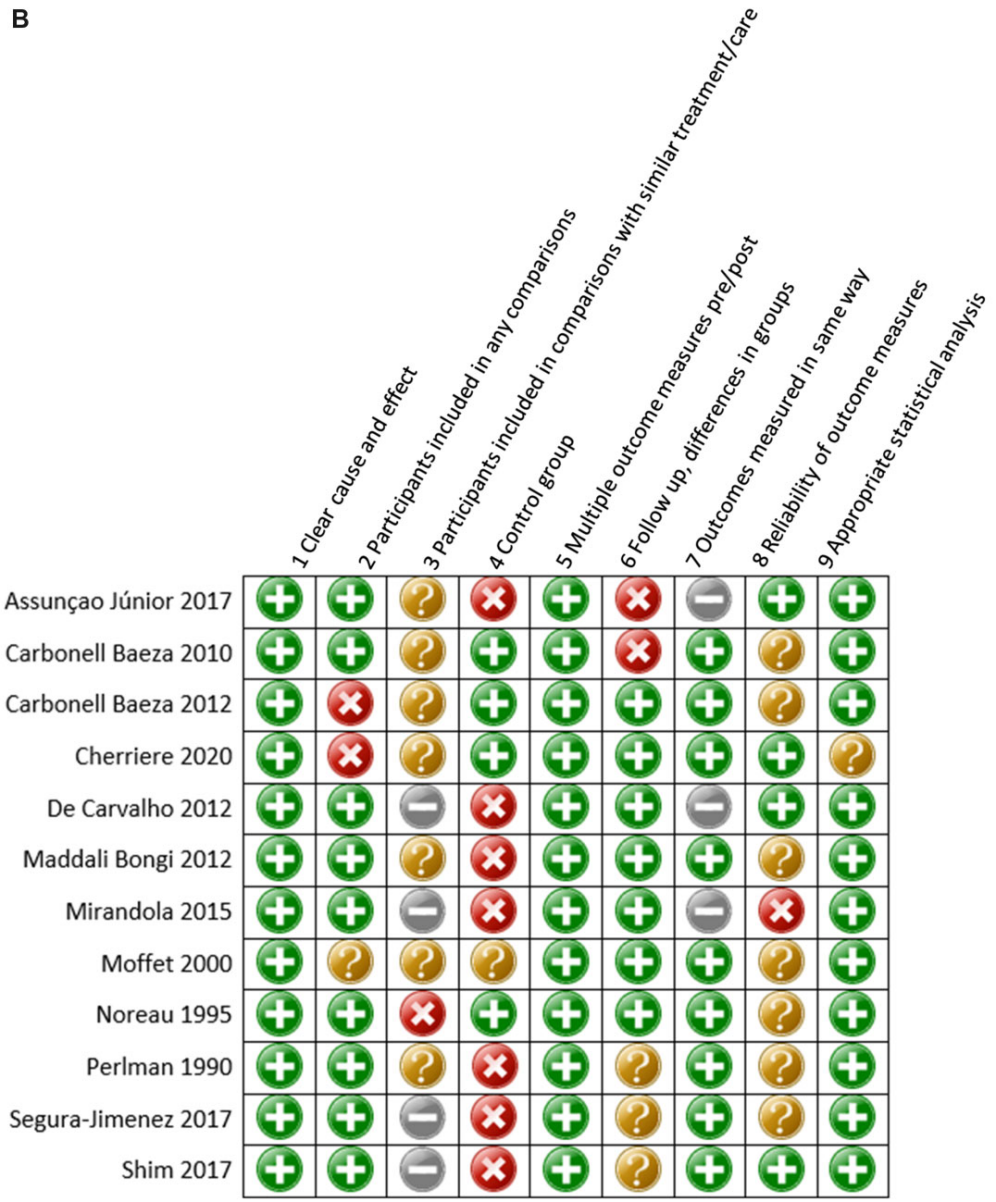

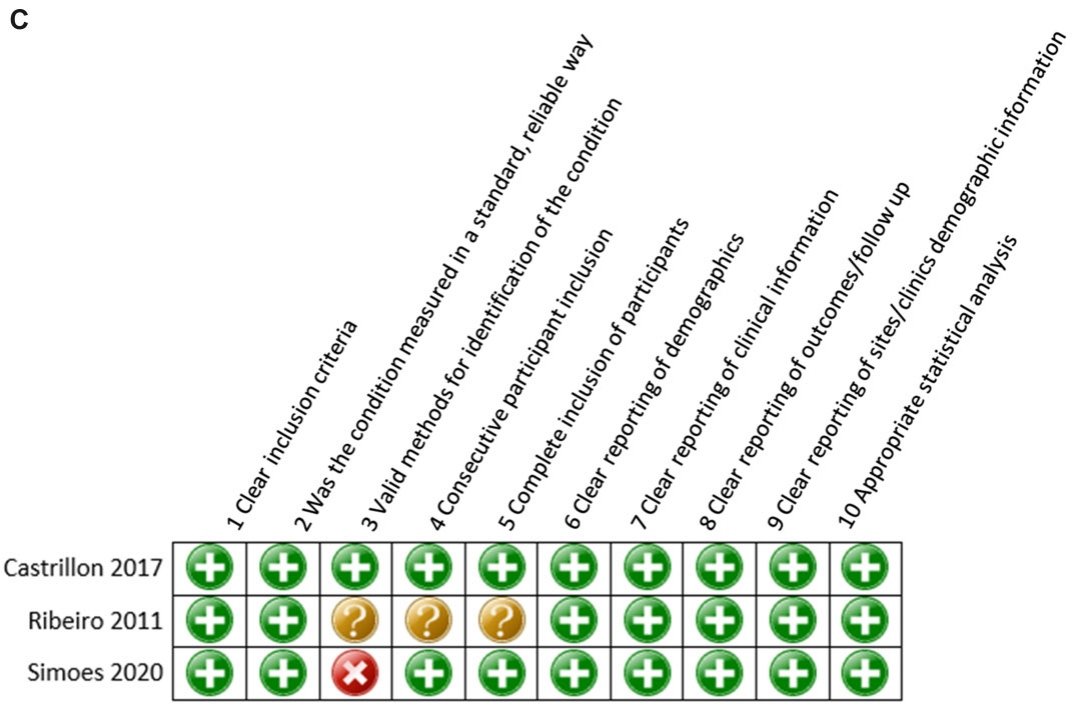

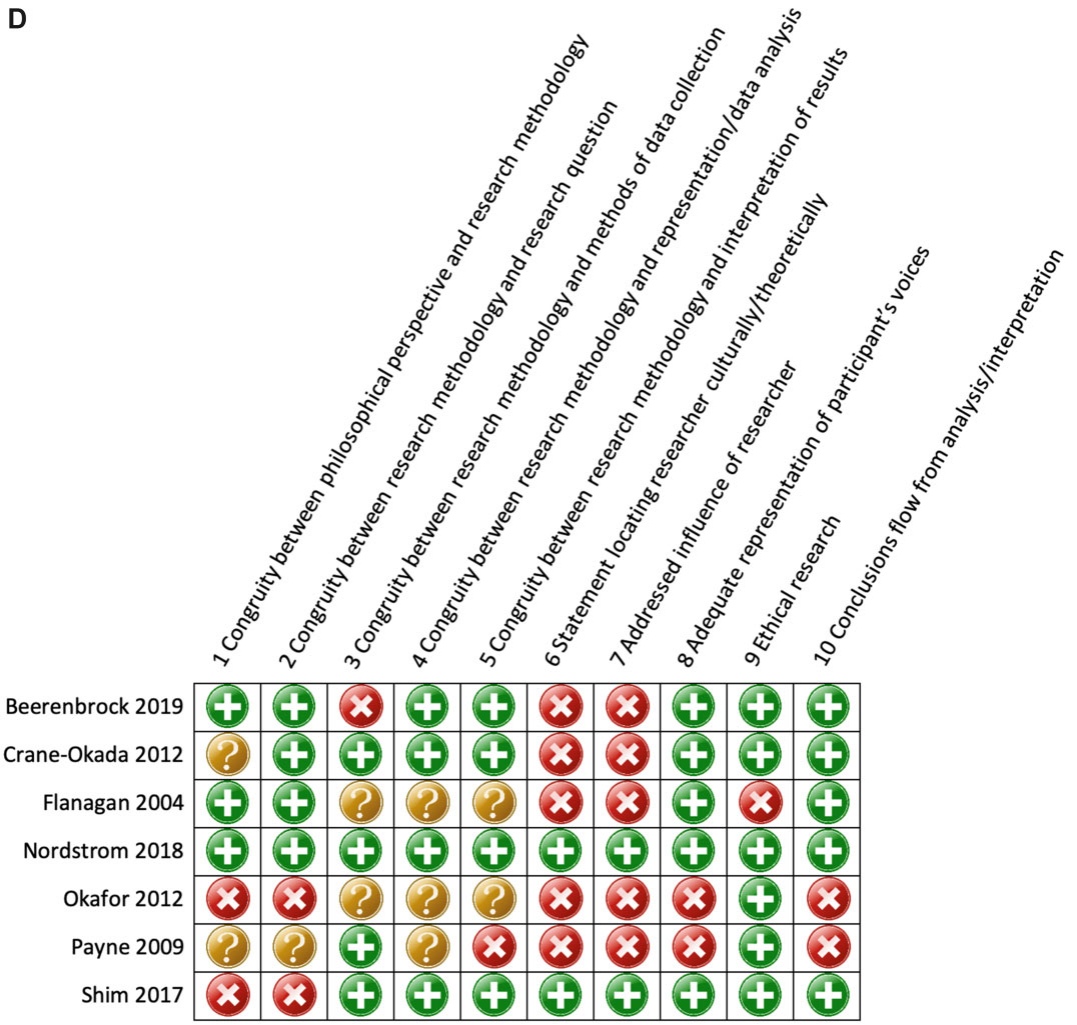
(A) Risk-of-bias assessment for randomized controlled trials (Joanna Briggs Institute Tool). **(B)** Risk-of-bias assessment for quasi-experimental studies (Joanna Briggs Institute Tool). **(C)** Risk-of-bias assessment for cases series (Joanna Briggs Institute Tool). **(D)** Risk-of-bias assessment for qualitative studies (Joanna Briggs Institute Tool).

Tallying scores on the JBI critical appraisal tools are discouraged [95]. Therefore, the following comments are made on general trends of bias across each of the study types. Randomized controlled trials had adequate standardization of outcome measures for control and intervention groups, appropriate statistical analysis, and trial design. However, the majority of studies had a lack of “true randomization, concealment of treatment allocation, assessor blinding and reliability of outcome measures” [56] (Figure 2A). Although it is not possible to blind therapists or participants to dance interventions, assessor blinding was not mentioned in any of the studies. Quasi-experimental studies all stated clear causes and effects, multiple outcome measures before and after the intervention, and appropriate statistical analysis (Figure 2B). Of the two case studies, one study [92] was highly robust and fulfilled all criteria, and another [93] had missing data around condition identification and complete and consecutive participant inclusion (Figure 2C). Critical appraisal of the qualitative studies was assessed via the JBI critical appraisal tool and found either a low risk of bias (n = 4) [61, 62, 64, 67] or a high risk (n = 3) [63,65,66] (Figure 2D). Four qualitative studies were performed with notable biases, including questionable “congruity between research methodology and representation and data analysis” [57], and lacked “statements locating the researcher culturally and addressing researcher influence” [57].

Overall, across all studies, 74% showed either a quantitative reduction in level of pain or qualitative themes of improvement in pain experience. Of the quantitative studies, 70.6% (n = 27) reported statistically significant improvements in at least one pain outcome measure. All mixed-methods studies [65–67] noted reduced pain quantitatively or described themes of improved coping or acceptance, whereas none of the case studies showed improvements in pain [92–94].

Qualitative themes of changing pain experience were taken only from the included qualitative studies and grouped together for narrative synthesis. The main qualitative themes included improved coping and acceptance of pain [64, 67], increased body understanding [61, 62, 64, 67], challenging fear of movement [62, 63, 67], acceptance of a new normalcy related to the participant’s chronic pain [61], new levels of mental and emotional well-being [61, 64, 65, 67], and freedom from pain [67].

Positive themes around pain or coping with pain [61–67] were found, of which dance therapy was the predominant dance genre used. Some women with breast cancer participating in a Mindful Movement Program found pain relief in the dance itself: “*I don’t feel the pain as much* *…* *when I’m dancing* *…* *it just kind of dissipates the pain*” [62]. Some women in the same program remarked on the effect of mindfulness and movement to help cope with pain: “*My mind-set would go into dealing with the pain, and the movement would help me with the pain, to deal with the pain*” [62]. For other participants within a private clinic, there was the belief that DMT physically and emotionally assisted reductions in pain: “*You are releasing pain and negative emotions out of your body* *…* *it helps you get out of the ‘stuck-ness’ or that mold you’ve been in*” [67]. Other ideas around improved pain coping were emphasized through themes of ordering chaos [63], creating new strategies [65], and achieving self-efficacy and resilience [67]. One participant attending a clinic program using DMT noted that “… *in my body the pain has changed from the worst thinkable to something I can live with* *…*” [63].

Although some participants found pain relief and the ability to cope with this pain after the dance intervention, others with increased pain chronicity found relief in their acceptance of the pain [61, 64, 67]. Acceptance of pain [64, 67] was the main benefit for some by refocusing on what was still possible for them: “*I am more accepting of my aging body and pain, because it helped me to realise that although I can’t do things that I used to do, I can find another way of doing things* *…*” [67]. Others found acceptance of their limitations and experienced a greater sense of normalcy and health, where researchers noted their tango intervention led to stronger acceptance of physical limits via awareness of musical and dancing abilities [61]. An understanding of limitations further allowed for greater interoception and consideration of bodily experiences.

Dance enabled an understanding of the body that was relayed through greater appreciation of sensing the body that was in pain: “[*Movement] made me aware of what did hurt and okay, it hurts, move it anyway*” [62]. Other participants found new meaning in body signals: “*To dance different parts of the body and then the feelings flared up* *…* *I didn’t have a clue that the pain and the feelings belonged together*” [64]. For those in a study participating in DMT, there was a broadening of thoughts and actions that led to learning “*new ways of living in the body and being in the world*” [67]. Further themes of participants regaining control of the body [61, 67] and taking responsibility for their own well-being were highlighted by Shim et al. (2017) [67]: “*I feel like I am in control, and can master the pain better* *…I don’t feel as helpless because there are things I can do to cope with it*” [67]. This new understanding of the body allowed for changes in the participant’s beliefs and perspectives.

A change in beliefs typically accompanied reductions in fear avoidance behavior and catastrophization, which helped to increase activity levels [67]. Similarly, participants in a DMT program challenged their fear of pain, which allowed them to find alternative ways to move [63]. Furthermore, those in a Mindful Movement Program reported less focus on the fear of pain reoccurrence in the future: “*I’m so much more in the here and now* *…* *Being able to step out of the craziness of the constantly being in the future*” [62]. By changing how participants related to their pain, they were able to change their response to the pain, which also includes their emotional responses to the pain.

There were a variety of themes around changed perception and outlook, such as the enhancement of emotional intelligence [67], connecting to self and others [67], connecting emotions and symptoms [65], feeling calmer and happier [64], and general positive feelings and sensations [61]. One participant in a DMT class noted the beneficial effects on his mental health: “*My attitude changed. It helped me to laugh and smile more, be kinder and open-minded*” [67]. A participant in a Free Movement Dance class found acceptance of his emotions through dance, noting that “*if you are sad you are sad* *…* *it is ok to feel whatever you feel; it is ok to be whoever you are*” [64].

Lastly, the ideas of freedom from pain [62] and motivation for movement [61] are important and were noted by Beerenbrock et al. (2020): “*I was able to let go and go with the flow and be more determined to be fluid in my postures and movement and dance*” [62]. Other observations were also noted by the researchers in a DMT study around disconnecting pain from identity: “… *by expressing their pain, pain-related thoughts and feelings in movement metaphors, participants were able to separate self from pain* *…*” (Shim et al., 2017) [67].

Whether pain was classified as chronic primary or chronic secondary musculoskeletal pain, the majority of all reviewed studies showed similar benefits from dance interventions when synthesizing both quantitative and qualitative data. There were improvements in pain experience and outcome measures, in both participants with chronic primary pain (n = 14 out of 16 studies) [63,64,66–68,70,71,75,76,81–83,85,90] (Table 3) and participants with chronic secondary musculoskeletal pain (n = 8 out of 10 studies) [61, 69, 72–74, 78, 79, 87–89]. Only one [65] of three studies classified as chronic neuropathic pain [84, 91, 93] noted improved pain measures, and both studies including chronic cancer treatment showed improved pain rating scores [86] and improved pain coping [62]. Lastly, one of three studies classified as chronic primary musculoskeletal pain showed improvements in pain measures [91].

Both categories of dance were beneficial in improving pain outcome measures or involved positive themes around the pain experience. Twelve out of the 20 articles using structured dance showed statistically significant reductions in pain [61, 65, 68, 69, 74, 78, 79, 85, 86, 88, 89, 91] or had themes of pain reduction [61, 65]. In comparison, all fourteen studies using dance therapies found statistically significant improvements in pain outcomes or themes of pain reduction, acceptance, or release. Nine studies used dance therapies that included quantitative measures, with seven reporting improvements in pain scores [67, 75, 76, 81–83, 90]. Studies with longer sessions of 90 minutes or more had longer intervention lengths of 16 weeks and were more likely to show significant improvements in pain outcome measures (80%) [56, 57, 75, 78] and themes of pain reduction and pain coping (100%) [60, 62, 70, 74] when compared with those 30–60 minutes long (71%).

An assessment of long-term follow-up was difficult, as only nine studies included an assessment after the end of the dance intervention [66, 68, 70–73, 81, 88, 92], averaging 17.8 weeks after the conclusion of the intervention, of which 66% maintained at least one statistically significant improvement in pain outcome [66, 68, 70, 71, 73, 81]. Studies with longer follow-ups of more than 13 weeks tended to maintain statistically significant improvements [68, 70, 71], but 75% of these studies were from those with fibromyalgia, which tends to be more likely to improve.

## Discussion

To our knowledge, this is the first systematic review to specifically investigate the literature around chronic pain and dance as a form of pain reduction and management and to encompass both quantitative and qualitative literature. Through this review, we identified promising research using dance to assist in pain reduction and improve qualitative psychosocial components of coping, self-efficacy, and pain acceptance across a variety of populations. There were overall improvements in pain identified from the narrative synthesis of both the quantitative and qualitative data that reflect a variety of positive experiences and changes in the participants. Dance therapies with durations of 60 minutes or longer appeared to show the greatest effect on pain management for those with chronic pain. The mechanisms of how these benefits might occur are speculated from this review and previous literature.

The narrative synthesis of the quantitative data from this review noted reductions in objective pain outcome measures over a range of participants with differing demographics and diagnoses partaking in a variety of dance interventions. Although pain was measured through a range of screening tools, 70.6% of studies using quantitative measures found statistically significant improvements in pain outcome measures. Furthermore, when synthesizing both quantitative and qualitative data relating to reduced pain perception, 74% of studies showed an improvement in pain. By triangulating the treatment effects with both quantitative and qualitative data, this offers insight into the multifaceted benefits of dance to not just improve the quantitative measures of pain but also address the complexity of the human experience of pain.

This review found numerous psychosocial benefits of dance, as reflected in the narrative synthesis of qualitative studies by the overall themes and voices of the individual participants. All studies that included qualitative data found at least one positive theme about pain management. Unexpectedly, many participants noted no decreased pain per se but rather improved acceptance of pain [64, 67], acceptance of a new normalcy [61], finding a new level of mental and emotional well-being [61, 64, 65, 67], or developing ideas around self-efficacy and resilience [67]. These qualitative aspects not only highlight the effect of dance on the lived experience of pain but also the value that these dance interventions have in changing perspectives and management of both pain and an individual’s overall health. Similarly, previous research has identified numerous psychosocial benefits for dance, including improved perceived emotional, physical, social, and spiritual dimensions [43], along with quality of life, depression, and anxiety [33]. Therefore, dance not only reduces the quantitative rating of pain but also has psychosocial benefits for individuals experiencing chronic pain and could therefore be a potent intervention for pain management.

The proposed components from all reviewed studies that are hypothesized to enable these benefits are physical activity, music, presence of a group setting, and physical touch. Our study found a variety of benefits in pain reduction and improved coping that could be attributed to serotonin and opioid secretion through exercise [96], descending pain modulation in the central nervous system when listening to music [97], and mirror neurons assisting in socially learned pain modulation [98]. This is relevant, as all the reviewed studies included a group setting, and those involving partnered touch [61, 62] showed their partner to assist with emotional and physical support of movement. Improvements in psychosocial parameters have been suggested to relate to functional changes that are associated with improved memory, attention, and psychosocial parameters as a result of dance [99]. The dance interventions included in the present review addressed the complex and multifaceted nature of pain and how it can be better managed.

This review found that the dance interventions that appeared to have the greatest influence on pain reduction and improved pain management were those with session durations of at least 60 minutes. Additionally, shorter-duration studies had fewer dropouts, of which those with 0% dropouts averaged intervention lengths of 7.7 weeks and those with 0–5% dropouts averaged intervention lengths of 9.4 weeks, potentially indicating better adherence with shorter study duration. Furthermore, interventions that approached the recommended 150 minutes of weekly activity [100] were more likely to show improved pain outcome measures; 86% of these showed improvements in quantitative measures or qualitative themes [65, 69, 72, 77–79, 89]. This is of importance to promote habitual physical activity [101], given that those with chronic pain generally do not meet the activity recommendation of moderate to vigorous activity for 150 minutes per week [102, 103]. As a result, we recommend that dance classes for those with chronic pain be a minimum of 60 minutes at least twice per week structured into 7- to 9-week blocks for positive results and to aid in adherence.

Although the majority of studies showed positive benefits for pain and the pain experience, some considerations should be noted in considering the results of this review. A number of qualitative and mixed-methods studies [61–67] recruited a convenience sample of participants who were current patients from the clinic running the study. This might have introduced a selection bias whereby these participants could have previous experience, rapport, trust, and belief in the treatment effect that are not reflective of the larger population. A majority of studies using dance therapies lacked clear reporting of methodology and were conducted poorly when assessed through the JBI risk-of-bias tool. This could reflect the exploratory nature of these interventions and a lack of adequate structure to warrant treatment standardization. The lack of reported compliance and adherence data leads to questions about the safety and potential risk of pain aggravation in those with chronic pain. However, this review found only one participant, out of 1,254 participants, who reported a pain flare-up, yet this was also accompanied by other benefits, such as awareness of body, regaining mobility, and feelings of pride and acceptance [61].

Although fibromyalgia is controversial as a diagnosis [104], we have included those with fibromyalgia as used in the included articles. Because of the large number (69%), robustness, and positive results from these studies, the results seen in the chronic primary pain category can be generalized only to those diagnosed with fibromyalgia. In contrast, studies of those classified as having chronic secondary musculoskeletal pain noted a variety of conditions, and as such they could be generalizable to the wider category population. Most study participants were women (75.2%), and as such, the results might not be generalizable to men; however, given this bias, it might also be expected that those with chronic pain participating in future dance programs could also be predominantly women [105]. Lastly, the lack of post-trial follow-up data across all studies leads us to question the long-term effects of these interventions on individuals with chronic pain and the likelihood of adherence to these interventions among individuals with chronic pain. As the original aim and criteria of this study were deliberately broad to capture any conditions with chronic pain, this lends itself to increased heterogeneity of study designs, comparison groups, populations, and interventions, making meta-analysis inappropriate. Nonetheless, the overall synthesis of both quantitative and qualitative data showed evidence for dance to reduce pain or improve participant coping across a variety of conditions.

Consideration of the individual dance class components would be beneficial in designing future dance interventions. Structured dance and dance therapy have different intentions: learning the steps and choreography of a dance style vs expressing oneself through movement and music, respectively. Therefore, structured dances could benefit from more creative and exploratory components, which have been noted in this review to have many psychosocial benefits. Dance therapy might benefit from more structure to the classes, such as adding in sections of choreography or technique and using more quantitative measures if using it as a tool for research. The creative component of dance therapy studies is commonly found solely in therapy-based dance practices and not in the wider dance community but could offer additional benefit for those experiencing chronic pain.

Other factors to be considered in future research and practice include reporting the music used, class formats, use of mirrors, and the use of touch between participants or the dance teacher [106]. The use of music is potent in its effects on pain and enjoyment and should be reported in any dance intervention. Some of these components, such as mirrors and touch, are typical and required in certain dance styles [107]. Both mirrors [108] and touch [109] have been effectively used in chronic pain management, and as such they could influence the experience of dance and chronic pain. Additionally, the presence of group movement and socialization before or after class could have positive benefits on pain experience [110]. Lastly, the results of this study should be considered in the context of group classes only, as we cannot comment on the effect of private one-on-one dance classes. Therefore, a greater understanding of class structure and use of external cues could influence the effectiveness of dance intervention for those experiencing chronic pain.

This review demonstrated a link between dance and the reduction of pain, with concurrent improved function. Notably, but outside the scope of this review, the reviewed studies showed that dance was more effective for pain management than were control interventions such as stretching [75, 76, 79], had an additional benefit when used as an adjunct to physiotherapy treatment [65], and was associated with improved functional outcomes as measured by questionnaire data [72, 75, 76, 81–83]. This demonstrates that dance could be a viable alternative or adjunct to current pain relief treatments, with additional benefits for improving function.

This review took a broad picture of the current literature on the use of dance to address pain in a variety of chronic pain populations. There appears to be value in the use of dance across a variety of pain-related conditions to reduce pain or improve pain coping in individuals with chronic pain, demonstrated through both quantitative and qualitative data. However, the lack of well-described and well-defined research specifically directed toward those with chronic pain conditions creates a gap between the current evidence base and the practical use of dance for pain management. This review supports the use of dance for those with chronic pain, with the recommendation that sessions be structured into 7- to 9-week blocks and a minimum of 60 minutes at least twice per week and include some creative components. Research in this field will further benefit from studies using both quantitative and qualitative methods with specific and detailed reporting of intervention, methodology, and results, which primarily investigates individuals with chronic pain.

## Notes

Funding sources: This research received no funding from any funding agency in the public, commercial, or not-for-profit sectors.

Study registration: International Prospective Register of Systematic Reviews: CRD42020165557.
